# Aptamer proteomics of serum exosomes from patients with Primary Raynaud’s and patients with Raynaud’s at risk of evolving into Systemic Sclerosis

**DOI:** 10.1371/journal.pone.0279461

**Published:** 2022-12-22

**Authors:** Sonsoles Piera-Velazquez, Simon T. Dillon, Xuesong Gu, Towia A. Libermann, Sergio A. Jimenez

**Affiliations:** 1 Jefferson Institute of Molecular Medicine, Scleroderma Center of Thomas Jefferson University, Philadelphia, Pennsylvania, United States of America; 2 Division of Interdisciplinary Medicine and Biotechnology, Beth Israel Deaconess Medical Center, Boston, Massachusetts, United States of America; 3 Genomics, Proteomics, Bioinformatics and Systems Biology Center, Beth Israel Deaconess Medical Center, Boston, Massachusetts, United States of America; 4 Harvard Medical School, Boston, Massachusetts, United States of America; NIH, UNITED STATES

## Abstract

**Background:**

A major unmet need for Systemic Sclerosis (SSc) clinical management is the lack of biomarkers for the early diagnosis of patients with Raynaud’s Phenomenon at high risk of evolving into SSc.

**Objective:**

To identify proteins contained within serum exosomes employing an aptamer proteomic analysis that may serve to reveal patients with Raynaud’s Phenomenon at risk of developing SSc.

**Methods:**

Exosomes were isolated from serum samples from patients with Primary Raynaud’s Phenomenon and from patients with Raynaud’s Phenomenon harbouring serum antinuclear antibodies (ANA) who may be at high risk of evolving into SSc. The expression of 1,305 proteins was quantified using SOMAscan aptamer proteomics, and associations of the differentially elevated or reduced proteins with the clinical subsets of Raynaud’s Phenomenon were assessed.

**Results:**

Twenty one differentially elevated and one differentially reduced (absolute fold change >|1.3|) proteins were identified. Principal component analysis using these 22 most differentially expressed proteins resulted in excellent separation of the two Raynaud’s Phenomenon clinical subsets. Remarkably, the most differentially elevated proteins are involved in enhanced inflammatory responses, immune cell activation and cell migration, and abnormal vascular functions.

**Conclusion:**

Aptamer proteomic analysis of circulating exosomes identified differentially elevated or reduced proteins between Raynaud’s Phenomenon at high risk of evolving into SSc and Primary Raynaud’s Phenomenon patients. Some of these proteins are involved in relevant biological pathways that may play a role in SSc pathogenesis including enhanced inflammatory responses, immune cell activation, and endothelial cell and vascular abnormalities.

## Introduction

Systemic Sclerosis (SSc) is a serious, disabling, and often fatal systemic autoimmune disease of unknown cause characterized by a severe and usually progressive cutaneous and visceral fibrotic process accompanied by extensive fibroproliferative vasculopathy and by humoral, and cellular immunity alterations [1–4]. One of the most common and earliest clinical manifestations of SSc is Raynaud’s Phenomenon [5–8]. However, Raynaud’s Phenomenon is also relatively common in the general population. Whereas Primary Raynaud’s Phenomenon is a benign condition usually evolving without any internal organ damage or increased mortality risk, Raynaud’s Phenomenon that may evolve into SSc clearly displays a more serious prognosis [5–8]. Therefore, there is a very important need to accurately establish which patients affected by Raynaud’s Phenomenon will remain as Primary Raynaud’s Phenomenon and which patients may be at risk of developing SSc before more serious clinical manifestations or internal organ involvement become apparent.

Although, there has been remarkable recent progress in the application of proteomic analysis of serum and other biological fluids for the identification of SSc biomarkers, most of these studies have employed mass spectrometry [9–11]. However, mass spectrometry involves complex and time consuming procedures and the overshadowing of proteins present at low concentrations by high abundance proteins represent strong limitations to its widespread application. The introduction of novel aptamer technologies that allow identification and simultaneous quantification of a large number of proteins with extremely high sensitivity and specificity across the entire dynamic range of protein concentrations may overcome these limitations. The remarkable increase in the sensitivity of aptamer proteomics and its high reproducibility in comparison to other more established and generally accepted proteomic platforms has been extensively demonstrated [12–15].

There has been recent interest in the characterization of proteins contained within secreted microvesicles for their potential as possible biomarkers for various diseases. Exosomes comprise a well-defined subtype of microvesicles released from all human cells into their surrounding intercellular space and into the circulation [16–20]. Exosomes are identified based on their size (30–150 nm or 30–200 nm), the mechanisms of their biogenesis and secretion from cells, and their unique macromolecule content that includes nucleic acids (DNA fragments, mRNAs, non-coding RNAs and miRNAs), proteins, and lipids [19–22]. It has been shown that the macromolecular content of exosomes depends on their cell of origin and, more importantly, reflects the functional or pathologic status of the cells [23]. Therefore, there has been great interest in the study of exosomal content as a source of potential diagnostic biomarkers for a variety of diseases, for the assessment of disease extent and severity, and to evaluate and monitor response to therapeutic interventions [21,22].

Owing to the extremely high sensitivity and specificity inherent in the developing field of aptamer proteomics, their application for the characterization and quantitative analysis of the protein content of serum and other biological fluids should be expected to yield highly valuable and novel disease biomarkers [24–27]. Indeed, some recent studies employed aptamer proteomics to analyze the proteomic content of serum from patients with various disorders including SSc [28,29]. These studies demonstrated a tremendous potential of aptamers for the discovery and identification of non-invasive biomarkers for numerous human disorders, and it is likely that aptamer proteomic analysis should continue to expand enhancing their value as diagnostic tools in the rapidly developing field of individualized medicine.

In the present study, we applied aptamer proteomic analyses of exosomes isolated from serum from patients with Raynaud’s Phenomenon who harbored in their serum a positive ANA test in comparison with patients with Primary Raynaud’s Phenomenon who had a negative ANA test. Our main objective was to identify differentially elevated or decreased proteins that may allow to separate patients who may be at high risk of developing SSc or who may be at the very early stages of SSc development [30].

## Materials and methods

### Population cohorts

The patients included in the study were enrolled and followed at the Scleroderma Center of Thomas Jefferson University (Philadelphia, PA, USA). There were 4 subjects with Raynaud’s Phenomenon with negative ANA and 3 subjects with Raynaud’s Phenomenon with positive ANA. All subjects were white females, their ages ranged from 21 to 53 years, and the duration of the Raynaud’s Phenomenon symptoms was from 1 to 11 years. The diagnosis of Raynaud’s Phenomenon was made based on the clinical manifestations presented by the subjects and on detailed physical examination. The diagnosis was in agreement with the criteria described in previous publications on Raynaud’s Phenomenon [5–7] and the International Consensus Criteria for the diagnosis of Raynaud’s Phenomenon [8]. At the time the samples were obtained for this study, none of the subjects had Sclerodactily, skin induration or other clinical manifestations of SSc. All patients also had serologic tests for a broad spectrum of autoantibodies. The serum ANA titers were 1:160 or greater in the cohort with positive ANA, and the patterns at the initial evaluation were anticentromere in one, speckled in another one, and homogeneous in the third subject. Of the relevance to the diagnosis of SSc was the fact that each of these patients developed Scl-70 antibodies at subsequent evaluations, thus providing strong support to the diagnosis of SSc in this cohort.

### Exosome isolation

Blood samples were obtained following approval of the Institutional Review Board (IRB). The IRB also approved the establishment of a serum biorepository at the Scleroderma Center (Protocol Title: “Biochemical and Vascular Alterations in Scleroderma”; IRB Protocol #06F.186). Serum samples were aliquoted and stored at -80°C until assayed. Exosomes were isolated from 0.5 ml of serum by size exclusion chromatography using the SBI (Scientific Bioscience, Palo Alto, CA.) SmartSEC HT EV Isolation System according to the manufacturer’s instructions. Essentially all of the microvesicles isolated employing this procedure are less than 200 nm according to the manufacturer’s brochure. For exosome protein extraction, the isolated exosomes were suspended in 1.0 ml of exosome buffer and proteins were extracted from 0.5 ml of each exosome sample using the M-PER extraction buffer (ThermoFisher Scientific, Watham, MA.) which yielded enough protein to proceed with SOMAscan analysis.

### SOMAscan assay

SOMAscan® analysis (SomaLogic, Inc., Boulder, CO) was performed in duplicate from samples of each patient included in the study. The analysis was performed at the BIDMC Genomics, Proteomics, Bioinformatics, and Systems Biology Center of Beth Israel Deaconess Medical Center (Boston, MA), an experienced SomaLogic certified service provider, according to standard protocols from SomaLogic that have been described elsewhere [31,32] using exosomes isolated at the Scleroderma Center of Thomas Jefferson University from the serum of the two Raynaud’s Phenomenon patient cohorts listed above. Briefly, using the recommended protocol from the manufacturer, 7.2 μg of sample of exosome protein lysate from each patient included in the study was run on the SOMAscan® Assay Kit for cell/tissue lysates 1.3k. This assay measures the concentration levels of 1,305 human proteins using highly selective single-stranded modified Slow Off-rate Modified DNA Aptamers (SOMAmer). Each individual protein concentration was transformed into a corresponding SOMAmer concentration, then quantified using a custom DNA microarray (Agilent) read-out which reports the data as relative fluorescence units (RFU), as described previously [31,32]. Three pooled normal serum exosome controls and one no-protein buffer control were run in parallel with the exosome test samples. Sample to sample variability was further controlled by several hybridization spike-in controls. Data quality control, calibration, and normalization were performed by SomaLogic according to the manufacturer’s protocol, as previously described [31,32]. All samples passed the SomaLogic standard quality control and normalization criteria for the manual 1.3k assay. Alterations in the expression of the identified proteins were analysed using the Student *t* test as described previously [31,32].

### Statistical analysis

Mean- and median fold-changes (FC) were calculated for proteins with statistically significant different concentration levels between the two Raynaud’s Phenomenon groups. Statistical significance was determined by using a *t*-test to compare SOMAscan® relative fluorescence units. Owing to the relatively small number of samples included in this study a protein was considered to be significantly elevated or decreased if the median absolute fold change was >|1.3| and the p-value was <0.1 as described previously [31,32]. Principal component analysis (PCA) was performed using XLSTAT (Addinsoft, Long Island City, NY) for the proteins with the largest increase or decrease in concentration to evaluate their ability to discriminate the two Raynaud’s Phenomenon clinical subsets.

### Systems Biology Analysis

To acquire new insights into potential pathophysiological pathways and biological functions underlying the exosomal protein signatures associated with the pathogenetic-difference or with the progression of patients with Raynaud’s Phenomenon and negative ANA to patients with Raynaud’s Phenomenon with positive ANA and to more precisely understand the complex interactions between the differentially increased or decreased proteins and potential candidate upstream regulators that may be involved in the progression or development of very early SSc, extensive Systems Biology Analysis was performed. These studies included assessment of functional category, upstream regulator, and regulator effect analyses of all dysregulated proteins with a p-value <0.1 and FC>|1.3| using the Ingenuity Pathway Analysis (IPA) software tool (QIAGEN, Redwood City, CA) as described previously [31,32]. The IPA software tool allows access to a repository of biological interactions and functions created from millions of individually modelled relationships ranging from the molecular (proteins, genes) to the organism (diseases) levels. The same top dysregulated proteins with a p-value <0.1 and FC>|1.3| were included in further network analysis using the STRING database version 11.0 for protein-protein functional and physical interactions, the results of which were displayed as a functional network. Interactions were considered with a medium confidence score of 0.4 or higher. Proteins without associations to other proteins in the network were removed. A k-means clustering algorithm was performed to select connected proteins (k-means = 3). Functional description of clusters was assigned based on a manually curated evaluation of enriched KEGG pathway, Gene Ontology (GO), Reactome, and STRING local network clusters terms.

## Results

### SOMAscan proteomics analysis

SOMAscan analysis comparing Raynaud’s Phenomenon with positive ANA to Primary Raynaud’s Phenomenon (with negative ANA) identified 22 dysregulated proteins with an absolute fold change of >|1.3| and a p value of <0.1 (21 increased, 1 decreased) as listed in **Table 1**.

**Table 1:** Top elevated (>1.30-fold and p value <0.1) and decreased (<1.30-fold and p value <0.1) proteins from serum exosomes of patients with Raynaud’s Phenomenon with positive ANA compared with serum exosomes from patients with Raynaud’s Phenomenon with negative ANA identified by aptamer proteomics.

Increased in Raynaud’s Phenomenon with Positive ANA					
SomaId	TargetFullName	Entrez Gene Symbol	p-val	Mean FC	Median Fc
SL000019	Apolipoprotein A-I (Apo A-1)	APOA1	0.045	5.66	7.18
SL004511	Bactericidal permeability-increasing protein (BPI)	BPI	0.065	2.76	2.89
SL000466	Insulin-like growth factor-binding protein 2 (IGFBP2)	IGFBP2	0.022	2.17	2.49
SL004009	Ras-related C3 botulinum toxin substrate 1 (RAC1)	RAC1	0.076	2.43	2.14
SL004516	Mannose-binding protein C (MBL)	MBL2	0.081	1.83	2.02
SL004765	MAP kinase-activated protein kinase 3 (MAPKAPK3)	MAPKAPK3	0.047	1.83	1.95
SL008039	Alcohol dehydrogenase [NADP(+)] (AK1A1)	AKR1A1	0.075	1.99	1.83
SL002782	Adrenomedullin	ADM	0.089	1.60	1.79
SL006542	Ficolin-2 (FCN2)	FCN2	0.053	1.60	1.72
SL003744	Galectin-3	LGALS3	0.035	2.03	1.71
SL000247	6-phosphogluconate dehydrogenase	PGD	0.077	1.53	1.60
SL000406	Eotaxin	CCL11	0.094	1.44	1.60
SL003522	Endoplasmic reticulum resident protein 29 (ERP29)	ERP29	0.094	1.39	1.57
SL018548	Alpha-1-antichymotrypsin complex	SERPINA3	0.050	1.42	1.49
SL004438	Cystatin-M	CST6	0.054	1.63	1.49
SL010384	Testican-1	SPOCK1	0.089	1.61	1.44
SL000617	Alanine aminotransferase 1 (ALT)	GPT	0.028	1.53	1.42
SL007173	Granulins (GRN)	GRN	0.023	1.35	1.35
SL003722	RAC-beta serine/threonine-protein Kinase (PKB beta)	AKT2	0.060	1.34	1.32
SL004556	Complement decay- accelerating factor (DAF)	CD55	0.086	1.29	1.31
SL005214	Semaphorin- 6A	SEMA6A	0.002	1.29	1.31
**Decreased in Raynaud’s Phenomenon with Positive ANA**					
**SomaId**	**TargetFullName**	**EntrezGeneSymbol**	**p-val**	**mean FC**	**Median Fc**
**SL000310**	**Complement C1r subcomponent (C1r)**	**C1R**	**0.035**	**-2.14**	**-1.93**

ANA: Serum antinuclear autoantibodies.

SomaId: SomaLogic identification number.

**Fig 1:**
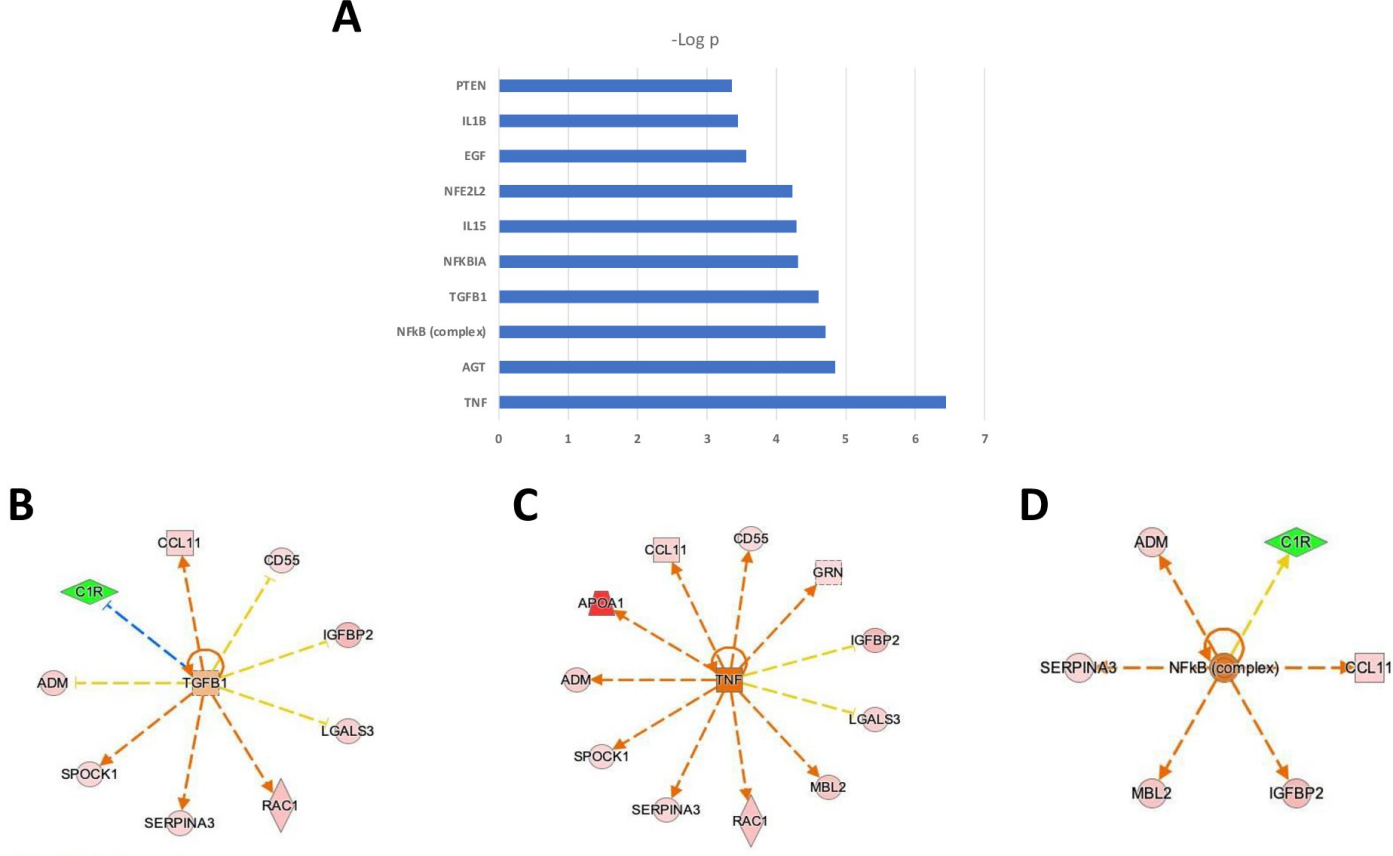
Principal component analysis of exosome proteins from patients with Raynaud’s Phenomenon with positive ANA compared to patients with Raynaud’s Phenomenon with negative ANA. Principal Component analysis of 22 differentially expressed proteins between samples of patients with Raynaud’s Phenomenon with negative ANA (green) and patients with Raynaud’s Phenomenon with a positive ANA (red).

Principal component analysis (PCA) using as input the top 22 differentially elevated or reduced proteins resulted in excellent separation of the two Raynaud’s Phenomenon cohorts, demonstrating that SOMAscan aptamer proteomics, indeed, identifies exosome proteins that clearly discriminate between the two clinical phenotypes as shown in **Fig 1**.

### Systems Biology Analysis of dysregulated pathways

**Fig 2:**
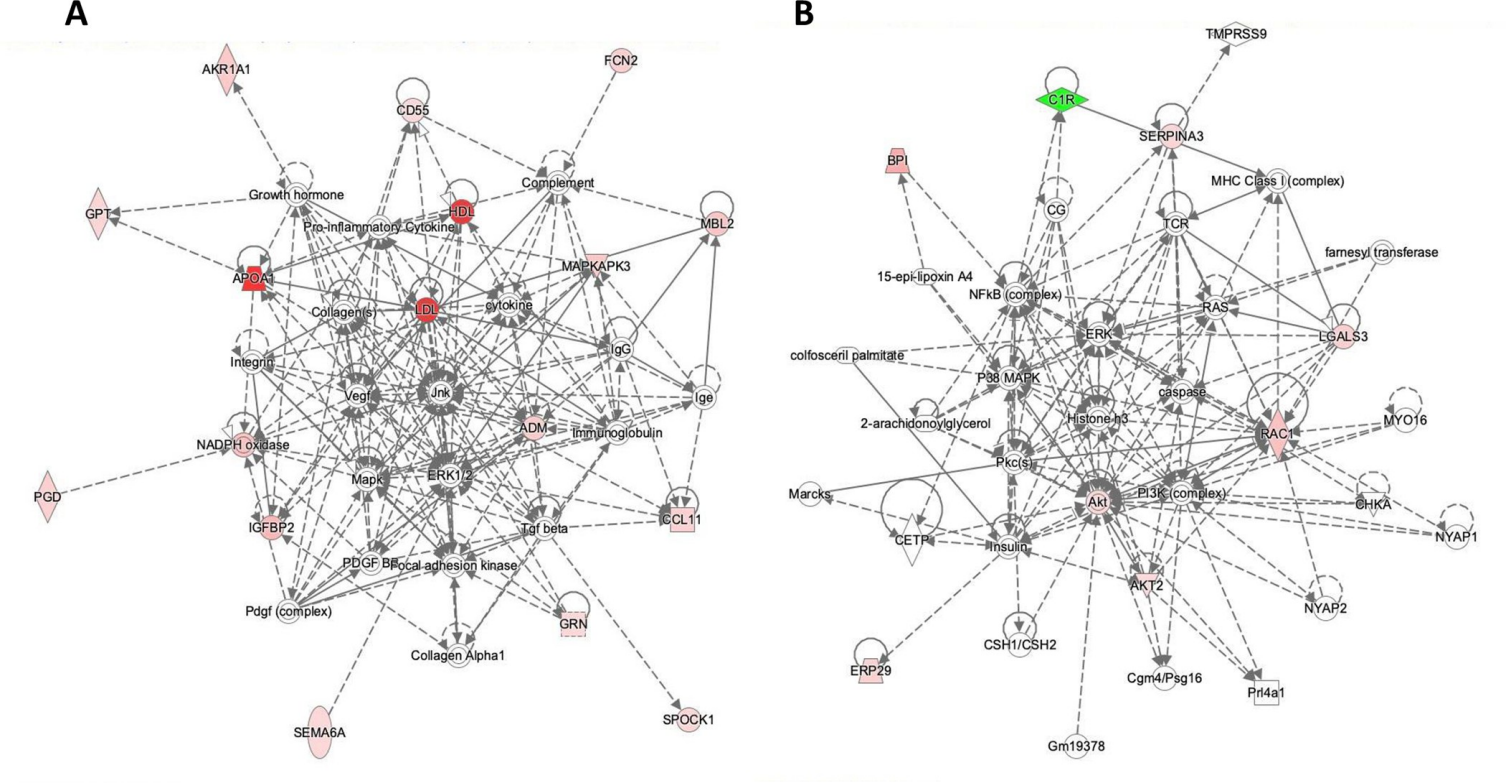
Biofunction analysis of exosome proteins from serum samples from patients with Raynaud’s Phenomenon with positive ANA compared to patients with Raynaud’s Phenomenon with negative ANA. A: Functional category. B-D: Biological Interactions: Inflammatory process (B); Immune response (C); and Endothelial cell and vascular involvement (D). IPA color and symbol guide: Blue = inhibition; orange = activation; red = increased; green = decreased; yellow = contrary to published evidence; grey = unknown; dashed line = indirect; arrowhead (pointed) = activating; arrowhead (blunt) = inhibitory. Proteins are coded by shape; square: Cytokine, vertical rhombus: Enzyme, horizontal rhombus: Peptidase, trapezoid: Transporter, ellipse: Transmembrane receptor, circle: Other.

To acquire new insights into potential pathophysiological pathways underlying the serum-derived exosome protein signature associated with ANA positive Raynaud’s Phenomenon, we performed functional category and upstream regulator analysis of the 22 dysregulated proteins with a p<0.1 and a fold difference >|1.3| using the Ingenuity Pathway Analysis (IPA) software tool (QIAGEN, Redwood City, CA). The Biofunction Analysis shown in **Fig 2** demonstrated that even among the relatively small number of samples used for analysis, the majority of the differentially expressed proteins were not a random set of proteins but were enriched for specific pathways (**Fig 2A**) and networks (**Fig 2B–2D**), showing clear biological relationships. Most prominent and with highest statistical significance of enrichment were pathways linked to vascular functions (Endothelial cell development; Proliferation of endothelial cells; Vasculogenesis), inflammation (Inflammation of body cavity; Inflammation of absolute anatomical region; Inflammation of organ), and myeloid cell movement and binding (Binding of professional phagocytic cells; Binding of myeloid cells; Cell movement of granulocytes; Cell movement of myeloid cells; Cell movement of phagocytes; Cell movement of neutrophils; Binding of monocytes).

**Fig 3:**
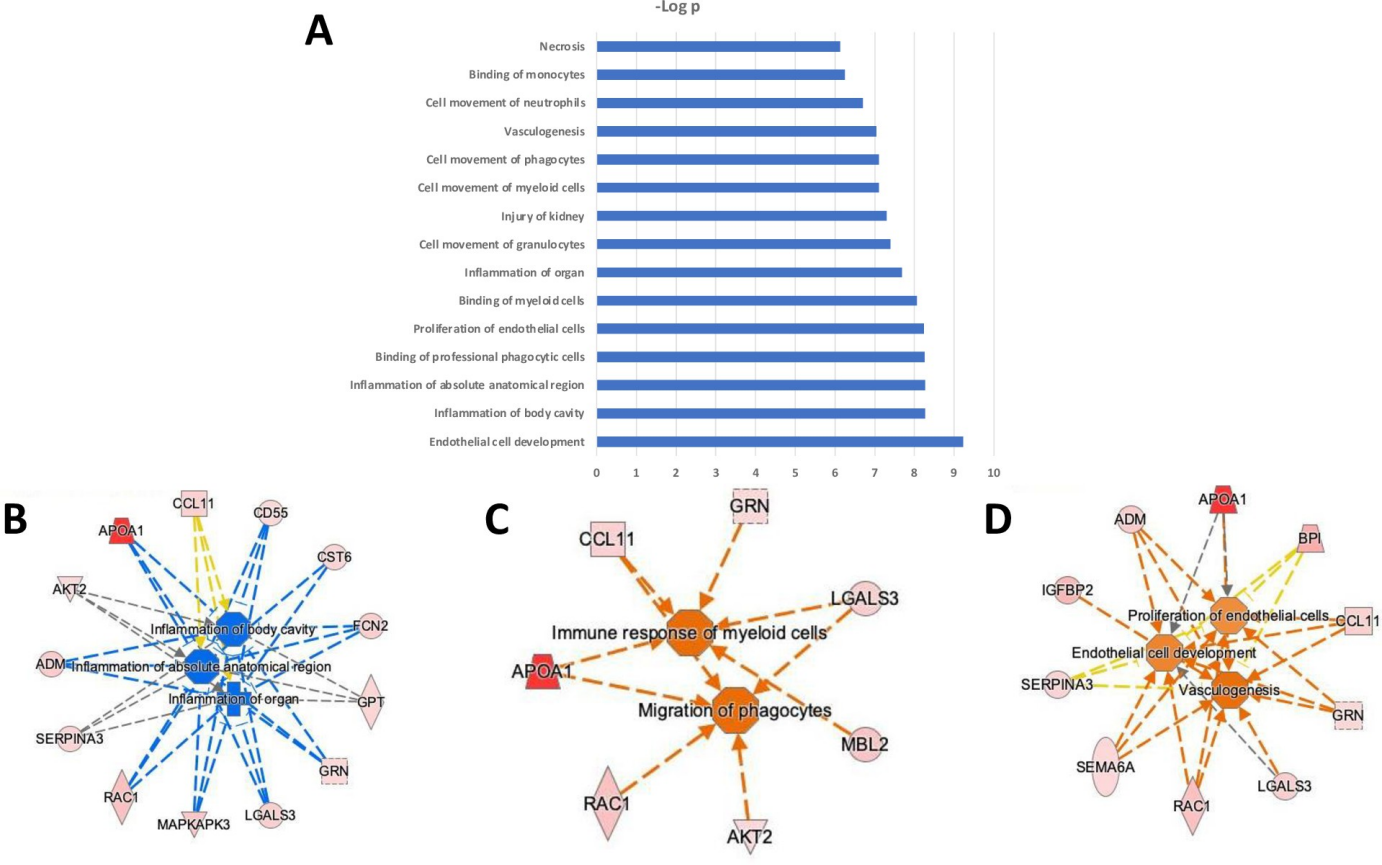
Upstream regulator analysis (A), downstream targets for TGFβ (B), for TNFα (C), and for NFΚβ (D) of exosome proteins from patients with Raynaud’s Phenomenon with positive ANA compared to patients with Raynaud’s Phenomenon with negative ANA. IPA color and symbol guide: Blue = inhibition; orange = activation; red = increased; green = decreased; yellow = contrary to published evidence; grey = unknown; dashed line = indirect; arrowhead (pointed) = activating; arrowhead (blunt) = inhibitory. Proteins are coded by shape; square: Cytokine, vertical rhombus: Enzyme, horizontal rhombus: Peptidase, trapezoid: Transporter, ellipse: Transmembrane receptor, circle: Other.

Especially informative was modelling the links between differentially expressed proteins based on their established connections with predicted upstream regulatory proteins. Upstream Regulator analysis predicted multiple proteins as potential regulators of these differentially elevated or reduced exosome proteins (**Fig 3A**). Most of the predicted upstream regulators with highest statistical significance for dysregulated proteins discriminating between Raynaud’s Phenomenon with positive ANA vs Primary Raynaud’s Phenomenon converged on activation of pro-inflammatory and immunoregulatory proteins (TNF0α, NFΚβ, NFKBIA, IL15) and extracellular matrix (TGFβ1) and vascular function regulators (AGT) (**Fig 3A**). These results indicated that these signalling nodes may be involved in the dysregulation of a sizeable portion of the top 22 proteins in the exosome proteomic signature for Raynaud’s Phenomenon with positive ANA compared with Primary Raynaud’s Phenomenon. Of substantial relevance in this regard were the observations that TGFβ1 regulated 9 out of the top 22 dysregulated proteins (**Fig 3B**), but remarkably, none of these were the typical profibrotic proteins associated with TGFβ. The results also showed that a key positive regulator of protein expression was the pro-inflammatory cytokine TNFα that was predicted to regulate 11 out of the top 22 dysregulated proteins (**Fig 3C**). These results strongly indicated that a potential increase in inflammatory processes may represent a crucial event that differentiates patients with Primary Raynaud’s Phenomenon from patients with the phenotype of Raynaud’s Phenomenon with positive ANA. This notion is further enhanced by the prediction of the pro-inflammatory transcription factor NFΚβ as a positive regulator of 6 of these proteins (**Fig 3D**) and was also reflected in the biological functions significantly enriched in Raynaud’s Phenomenon with positive ANA compared with Primary Raynaud’s Phenomenon. These biological function included predicted enhanced migration, movement, binding, and immune response of myeloid cells, granulocytes, and phagocytes linked to 11 proteins (**Fig 2**). Ten proteins were also predicted to enhance vasculogenesis and endothelial cell proliferation (**Fig 2A and 2D**) and a regulator model predicted by IPA highlighted TGFβ and TNFα as positive upstream regulators of numerous proteins that are anticipated to modulate vasculogenesis (**Fig 3B and 3C**).

**Fig 4:**
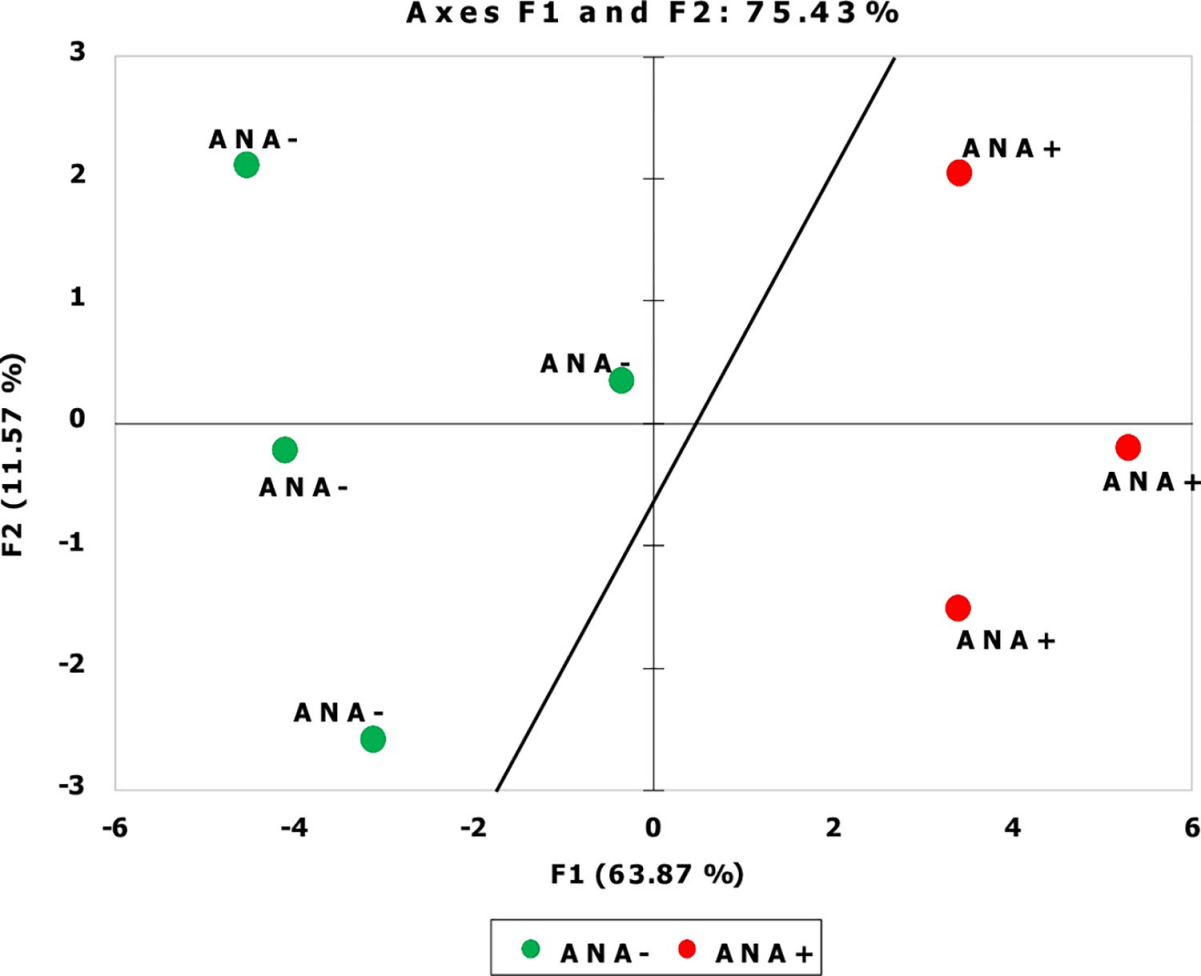
STRING analysis of exosome proteins from patients with Raynaud’s Phenomenon with positive ANA compared to patients with Raynaud’s Phenomenon with negative ANA. Protein–protein interaction network was created based on analysis of 12 proteins out of 22 proteins from the comparison of samples with Raynaud with positive ANA vs Raynaud with negative ANA. k-means  =  3 clusters indicated by node color. Solid line represents within-cluster, dashed lines represent between-cluster interactions.

**Fig 5:**
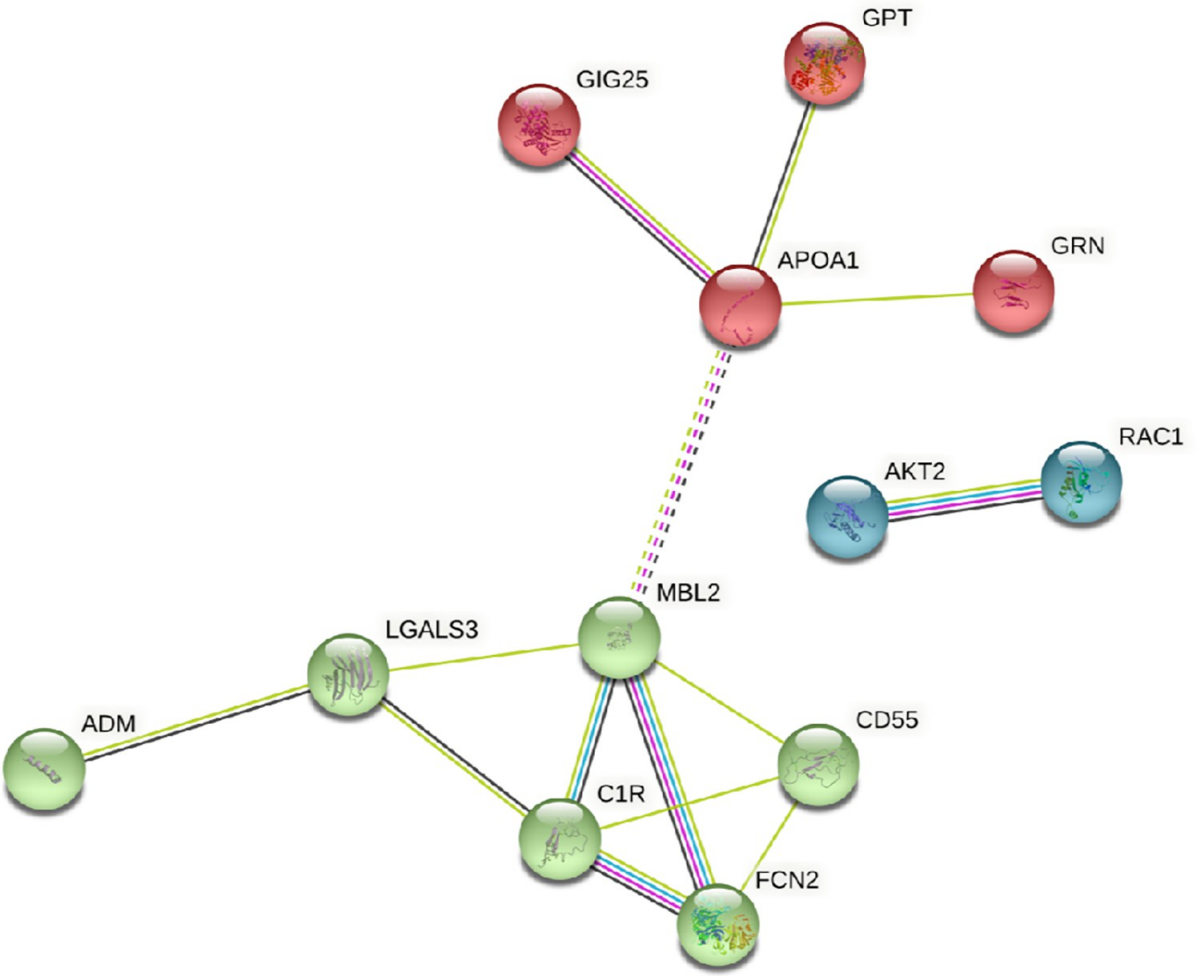
Networks of interacting proteins from exosomes isolated from patients with Raynaud’s Phenomenon and positive ANA compared with exosomes from patients with Raynaud’s Phenomenon and negative ANA. A: Network of JNK and ERK interactions; B: Network of AKT and RAC1 interactions. IPA color and symbol guide: Blue = inhibition; orange = activation; red = increased; green = decreased; yellow = contrary to published evidence; grey = unknown; dashed line = indirect; arrowhead (pointed) = activating; arrowhead (blunt) = inhibitory. Proteins are coded by shape; square: Cytokine, vertical rhombus: Enzyme, horizontal rhombus: Peptidase, trapezoid: Transporter, ellipse: Transmembrane receptor, circle: Other.

Next, we performed network and cluster analysis using the STRING database of functional and physical protein associations curated across major data repositories (**Fig 4**). Twelve of the 22 proteins discriminating between Raynaud’s Phenomenon with positive ANA and Primary Raynaud’s Phenomenon formed distinct interacting protein clusters with links to immune response and the complement system (**Fig 4**). An alternative interactive network analysis was performed using as input the most differentially expressed proteins from the comparison between the two Raynaud’s Phenomenon subsets using IPA that also incorporates genes/proteins that are not differentially expressed but directly connected. The results showed that the largest number of the differentially elevated proteins from the samples of patients with Raynaud’s positive ANA are predicted to participate in JNK/ERK and AKT/RAC1 networks as shown by these proteins as focus hubs. These focus hubs contain proteins with the most connections, that are thought to be functionally critical for the network and whose interruption would be expected to have a substantial/profound biological impact as shown in **Fig 5**.

## Discussion

One of the most common and earliest clinical manifestations of SSc is Raynaud’s Phenomenon [5–7]. A large study by Koenig et al. indicated that Raynaud’s Phenomenon associated with the presence of swollen or puffy fingers and a positive serum ANA was predictive of the progression to SSc [30]. Since the appearance of a positive ANA test in the serum may be slow to develop, it is crucially important to identify earlier biomarkers that may help to accurately establish which patients affected by Raynaud’s Phenomenon may be at risk of developing SSc before more serious clinical manifestations or internal organ involvement become apparent or before they develop clinical and pathologic SSc alterations. At the same time, identification of early disease biomarkers is critical to better understand the pathophysiological processes involved in the development of SSc clinical manifestations and to provide new insights into therapeutic strategies for prevention or delay of SSc development.

A highly promising new proteomics technology, SOMAscan, for the analysis and quantification of protein biomarkers employs chemically modified aptamers. Aptamers are RNA molecules that exhibit highly complex molecular recognition properties and are capable of binding specifically and with high affinity to targets ranging from small molecules to complex multimeric structures [12–15]. Aptamers have a remarkable dynamic detection range encompassing femtomolar to micromolar concentrations. Owing to its very high sensitivity it is generally expected that aptamer proteomic technology should enable the quantitative assessment of proteins present at very low concentrations in samples from various biological fluids as well as to assay for a large number of proteins simultaneously. The aptamer-based proteomic analysis utilizing the SOMAscan assay is a highly multiplexed, sensitive, quantitative and reproducible proteomic platform that offers excellent reproducibility and a low coefficient of variability [12–15]. The SOMAscan assay employed in the current study measures the levels of 1,305 analytes over a wide dynamic range (>10 logs of concentration) with minimal amounts of protein needed (7.2 μg of exosomal proteins).

One of the most promising and novel approaches for the discovery of disease biomarkers is the analysis of exosomes that are isolated and purified from serum, plasma, and other biological fluids. Exosomes are a subtype of extracellular vesicles (EV) that contain a diverse array of macromolecular species, including multiple forms of nucleic acids (mRNAs, miRNAs and various other non-coding RNAs), proteins (cytokines, chemokines, growth factors and transcription factors), lipids, and other metabolites [16–22]. Since the macromolecular content of exosomes reflects the cells from which they were released, as well as any functional, pathological or phenotypic changes occurring in these cells [23], the analysis of their macromolecular content would be expected to yield biomarkers that serve not only as valuable diagnostic tools for various diseases but also as indicators of disease improvement or progression. Indeed, several studies have described the application of exosome proteomics to identify potential biomarkers for numerous diseases [Reviewed in 19–22]. Moreover, two studies showed that exosomes were carriers of pro-fibrotic signals [33,34], or that they may function as messengers between the immune, vascular and fibrotic components in SSc [35]. Despite this promising potential approach, few aptamer proteomic studies applied to SSc have been described [28,29]. However, these published studies [28,29], did not examine the proteomic content of isolated SSc serum exosomes.

The results obtained in the current exploratory study identified 22 proteins that were differentially elevated or decreased in the comparative analysis of the two sets of Raynaud’s Phenomenon patients that had not been previously considered to be associated with SSc development or pathogenesis. Remarkably, some of these proteins display inflammatory, immunologic and vascular effects. Among these proteins, Apolipoprotein A-1 (Apo A-1) was the highest differentially elevated protein in the exosomes from patients with Raynaud’s Phenomenon with positive ANA (7.18-fold). Although Apo A-1 is well known to play a crucial role in maintenance of high density lipoproteins and is recognized as playing a protective role in various cardiovascular events including myocardial infarction, several recent studies have pointed out that Apo A-1 may also participate in the regulation and development of inflammatory events [36]. Another protein that was differentially elevated was the mannose binding protein (MBL2), a liver-based mannose-binding lectin that is a significant constituent of the human innate immunity and appears to be a major regulator of inflammation [37]. There was also a nearly three-fold elevation of the bactericidal/permeability-increasing protein (BPI). BPI is a lipid-transfer protein mainly present in neutrophils, and to a lesser extent in eosinophils that is endowed with potent and selective antimicrobial and endotoxin-neutralizing properties. Although, a potential role for BPI in the pathogenesis of autoimmune disorders has not been described, it has been shown that there is a high prevalence of autoantibodies against BPI (BPI-ANCA) in serum from patients with various infectious, inflammatory, and rheumatic diseases [38], and that its overexpression may induce inflammatory events in patients with SLE [39]. Furthermore, BPI has been shown to inhibit angiogenesis and cause endothelial cell apoptosis [40].

Several other proteins of interest will be briefly discussed in the following paragraphs. One of these is IGFBP-2. It has recently been demonstrated that IGFBP-2 may display numerous activities not related to insulin binding, including some effects on profibrotic and proinflammatory pathways. Indeed, the serum levels of IGFBP-2 were shown to be elevated in SSc patients with lung vascular involvement compared to healthy donors, and of remarkable importance were observations that IGFBP-2 levels displayed a negative correlation with pulmonary function in these patients [41]. Another protein is RAC1, the small GTPase Ras-related C3 botulinum toxin substrate 1. RAC1 plays a central role in skin homeostasis, including barrier function, wound healing and inflammatory responses [42]. RAC1 is also involved in the maintenance and stabilization of microvascular endothelial barrier functions and is essential for wound re-epithelialization, therefore, its activity is increased during wound healing [42]. It has also been shown that RAC1 Inhibition reduces the fibrotic phenotype of activated fibroblasts [43].

Mitogen-activated protein kinases (MAPKs) family of enzymes regulates a diverse array of physiological processes including cell proliferation and differentiation, development, immune function, stress responses, and cell death. The MAPK-activated kinase 3 (MAPKAPK3) also has been implicated in the inflammatory response [44]. Of substantial interest were the results with Galectin-3 (Gal3) which is a member of the β-galactoside-binding lectin family mainly found in epithelial and myeloid cells but also found in other cell types including a variety of immune cells. Gal3 binds various cell receptors and extracellular matrix proteins and selectively mediates key intracellular signaling pathways leading to the release of several cytokines. Numerous biological functions where Gal3 plays a mediator role have been recognized, including regulation of extracellular matrix and epithelial cell interactions, cell growth and differentiation, regulation of immune surveillance, angiogenesis and proinflammatory activities. Recent evidence indicates that Gal3 plays an important role in the pathogenic mechanisms of tissue fibrosis by increasing fibroblast proliferation and collagen production [45]. Also, some studies showed higher serum levels of Gal3 in SSc patients compared with normal donors and described Gal1 and Gal3 associations with SSc clinical features [46,47]. However, in another study Gal1 and Gal3 expression levels were found to be lower in SSc lesional skin compared with normal donor skin [48].

Finally, some studies have examined the levels of α1-antichymotrypsin, also known as SERPIN3A. SERPIN3A protein is an acute-phase reactant produced by the liver. Its biologic function is to inhibit several serine proteases, mainly cathepsin G, which is contained in the neutrophil granules and released at sites of inflammation. Notably, an excess of cathepsin G function is linked to tissue damage. Other interesting proteins included among the SERPIN3A and Lectin pathway proteins have been implicated in SSc and elevated serum levels are associated with renal crisis [49,50].

The novelty of the studies reported here lies in the application of aptamer proteomics for identification of differentially increased or decreased proteins in extracts from exosomes isolated from the serum of patients with Primary Raynaud’s Phenomenon with negative ANA compared with patients with Raynaud’s Phenomenon with positive ANA. This later population is considered to be at high risk of evolving into a systemic autoimmune disease, most likely SSc. Although we acknowledge and are very aware that the number of patients included in the Raynaud’s Phenomenon cohorts studied here is quite small representing a serious weakness to this study, we believe that the description of the results obtained with samples from these patients may provide a strong stimulus to perform subsequent studies that may pursue the study of the novel proteins in SSc pathogenesis and also to identify useful and valuable biomarkers for the diagnosis of patients with Raynaud’s Phenomenon at high risk of evolving into SSc.

We further recognize that the results described here will require validation in larger populations of patients with Raynaud’s Phenomenon to confirm and demonstrate their clinical utility. However, it is important to emphasize that, currently, there is a serious lack of validated biomarkers that may allow to identify SSc patients at the earliest stages of SSc development. This represents an important unmet need since given the serious clinical implications of SSc it is of crucial importance to establish an early diagnosis that may allow the institution of appropriate therapeutic measures that may be effective to prevent further progression to the development of the full SSc clinical manifestations and high mortality rate. Furthermore, the results we obtained identified numerous proteins that were differentially elevated in the samples from ANA + Raynaud’s Phenomenon patients that had not been previously associated with SSc pathogenesis but that as discussed above participate in important cellular functions that may be of great relevance to the pathogenesis of SSc and, therefore, may open the gate for extensive future investigation to establish the role of these newly identified proteins in SSc development and pathogenesis.

## References

[1] Varga J, Abraham D. Systemic sclerosis: a prototypic multisystem fibrotic disorder. J Clin Invest. 2007;117:557–567. doi: 10.1172/JCI31139 17332883 PMC1804347

[2] Gabrielli A, Avvedimento EV, Krieg T. Scleroderma. N Engl J Med. 2009;360:1989–2003. doi: 10.1056/NEJMra0806188 19420368

[3] Allanore Y, Simms R, Distler O, Trojanowska M, Pope J, Denton CP, et al. Systemic sclerosis. Nat. Rev. Dis. Prim. 2015; 1: 15002. doi: 10.1038/nrdp.2015.2 27189141

[4] Denton C.P., Khanna D. Systemic sclerosis. Lancet. 2017;390:1685–99. doi: 10.1016/S0140-6736(17)30933-9 28413064

[5] Herrick AL. The pathogenesis, diagnosis and treatment of Raynaud phenomenon. Nat Rev Rheumatol. 2012; 8:469–479. doi: 10.1038/nrrheum.2012.96 22782008

[6] Wigley FM, Flavahan NA. Raynaud’s Phenomenon. N Engl J Med. 2016;375:556–565. doi: 10.1056/NEJMra1507638 27509103

[7] Pauling JD, Hughes M, Pope JE. Raynaud’s phenomenon-an update on diagnosis, classification and management. Clin Rheumatol. 2019;38(12):3317–3330. doi: 10.1007/s10067-019-04745-5 31420815

[8] Maverakis E, Patel F, Kronenberg DG, Chung L, Florentino D, Allanore Y, et al. International consensus criteria for the diagnosis of Raynaud’s phenomenon. J Autoimmun. 2014; 48–9:60–65. doi: 10.1016/j.jaut.2014.01.020 24491823 PMC4018202

[9] Castro SV, Jimenez SA. Biomarkers in systemic sclerosis. Biomark Med. 2010;4:133–147. doi: 10.2217/bmm.09.79 20387310 PMC3049114

[10] Castelino FV, Varga J. Current status of systemic sclerosis biomarkers: applications for diagnosis, management and drug development. Expert Rev Clin Immunol. 2013;9:1077–1090. doi: 10.1586/1744666X.2013.848792 24168414

[11] Wermuth PJ, Piera-Velazquez S, Rosenbloom J, Jimenez SA. Existing and novel biomarkers for precision medicine in systemic sclerosis. Nat Rev Rheumatol. 2018;14(7):421–432. doi: 10.1038/s41584-018-0021-9 29789665

[12] Ellington AD, Szostak JW. In vitro selection of RNA molecules that bind specific ligands. Nature 1990;346:818–822. doi: 10.1038/346818a0 1697402

[13] Tuerk C, Gold L. Systematic evolution of ligands by exponential enrichment: RNA Ligands to bacteriophage T4 DNA polymerase. Science 1990;249:505–510. doi: 10.1126/science.2200121 2200121

[14] Kraemer S, Vaught JD, Bock C, Gold L, Katilius E, Keeney TR, et al. From SOMAmer-based biomarker discovery to diagnostic and clinical applications: a SOMAmer-based, streamlined multiplex proteomic assay. PLoS One. 2011;6(10):e26332. doi: 10.1371/journal.pone.0026332 22022604 PMC3195687

[15] Gold L, Walker JJ, Wilcox SK, Williams S. Advances in human proteomics at high scale with the SOMAscan proteomics platform. Nature Biotechnol 2012;29:543–549. doi: 10.1016/j.nbt.2011.11.016 22155539

[16] Thery C, Zitvogel L and Amigorena S, Exosomes: composition, biogenesis and function, Nature reviews. Immunology. 2002; 2:569–579. doi: 10.1038/nri855 12154376

[17] Vlassov AV, Magdaleno S, Setterquist R, Conrad R. Exosomes: current knowledge of their composition, biological functions, and diagnostic and therapeutic potentials. Biochem Biophys Acta. 2012;1820:940–948. doi: 10.1016/j.bbagen.2012.03.017 22503788

[18] Colombo M, Raposo G, Théry C. Biogenesis, secretion, and intercellular interactions of exosomes and other extracellular vesicles. Annu Rev Cell Dev Biol 2014;30:255–289. doi: 10.1146/annurev-cellbio-101512-122326 25288114

[19] Pegtel DM, Gould SJ. Exosomes. Annu Rev Biochem. 2019 Jun 20;88:487–514. doi: 10.1146/annurev-biochem-013118-111902 31220978

[20] Kalluri R, LeBleu VS. The biology, function, and biomedical applications of exosomes. Science. 2020; 367(6478): eaau6977. doi: 10.1126/science.aau6977 32029601 PMC7717626

[21] Simpson RJ, Lim JW, Moritz RL, Mathivanan S. Exosomes: proteomic insights and diagnostic potential. Expert Rev Proteomics. 2009;6:267–283. doi: 10.1586/epr.09.17 19489699

[22] Raimondo F, Morosi L, Chinello C, Magni F, Pitto M. Advances in membranous vesicle and exosome proteomics improving biological understanding and biomarker discovery. Proteomics. 2011;11:709–720. doi: 10.1002/pmic.201000422 21241021

[23] Willms E, Johansson HJ, Mäger I, Lee Y, Blomberg KE, Sadik M, et al. Cells release subpopulations of exosomes with distinct molecular and biological properties. Sci Rep. 2016;6:22519. doi: 10.1038/srep22519 26931825 PMC4773763

[24] Wilson R. High-content aptamer-based proteomics. J Proteomics. 2011; 74:1852–1854. doi: 10.1016/j.jprot.2011.04.017 21980599

[25] Janssen KP, Knez K, Spasic D, et al. Multiplexed protein detection using an affinity aptamer amplification assay. Anal Bioanal Chem. 2012; 404:2073–2081. doi: 10.1007/s00216-012-6252-8 22825678

[26] Yoshida Y, Waga I, Horii K. Quantitative and sensitive protein detection strategies based on aptamers. Proteomics Clin Appl. 2012; 6:574–580. doi: 10.1002/prca.201200037 22996907

[27] Huang J, Chen X, Fu X, Li Z, Huang Y, Liang C. Advances in Aptamer-Based Biomarker Discovery. Front Cell Dev Biol. 2021;9:659760. doi: 10.3389/fcell.2021.659760 33796540 PMC8007916

[28] Rice LM, Mantero JC, Stifano G, Ziemek J, Simms RW, Gordon J et al., A Proteome-Derived Longitudinal Pharmacodynamic Biomarker for Diffuse Systemic Sclerosis Skin. J Invest Dermatol. 2017;137(1):62–70. doi: 10.1016/j.jid.2016.08.027 27640094

[29] Rice LM, Mantero JC, Stratton EA, Warburton R, Roberts K, Hill N, et al. Serum biomarker for diagnostic evaluation of pulmonary arterial hypertension in systemic sclerosis. Arthritis Res Ther. 2018;20(1):185. doi: 10.1186/s13075-018-1679-8 30115106 PMC6097341

[30] Koenig M, Joyal F, Fritzler MJ, Rousin A, Abrahamowicz M, Boire G, et al. Autoantibodies and microvascular damage are independent predictive factors for the progression of Raynaud’s phenomenon to systemic sclerosis: a twenty-year prospective study of 586 patients, with validation of proposed criteria for early systemic sclerosis. Arthritis Rheum. 2008;58:3902–3912. doi: 10.1002/art.24038 19035499

[31] Larson A, Libermann TA, Bowditch H, Das G, Diakos N, Huggins GS, et al. Plasma Proteomic Profiling in Hypertrophic Cardiomyopathy Patients before and after Surgical Myectomy Reveals Post-Procedural Reduction in Systemic Inflammation. Int J Mol Sci. 2021;22(5):2474. doi: 10.3390/ijms22052474 33804404 PMC7957543

[32] Kim CH, Tworoger SS, Stampfer MJ, Dillon ST, Gu X, Sawyer SJ, et al. Stability and reproducibility of proteomic profiles measured with an aptamer-based platform. Sci Rep. 2018;8(1):8382. doi: 10.1038/s41598-018-26640-w 29849057 PMC5976624

[33] Wermuth PJ, Piera-Velazquez S, Jimenez SA. Exosomes isolated from serum of systemic sclerosis patients display alterations in their content of profibrotic and antifibrotic microRNA and induce a profibrotic phenotype in cultured normal dermal fibroblasts. Clin Exp Rheumatol. 2017; 35:21–30. 28094758 PMC6530475

[34] Li M, Jiang M, Meng J, Tao L. Exosomes: Carriers of Pro-Fibrotic Signals and Therapeutic Targets in Fibrosis. Curr Pharm Des. 2019; 25: 4496–4509. doi: 10.2174/1381612825666191209161443 31814552

[35] Colletti M, Galardi A, De Santis M, Guidelli GM, Di Giannatale A, Di Luigi L, et al. Exosomes in Systemic Sclerosis: Messengers Between Immune, Vascular and Fibrotic Components? Int. J. Mol. Sci. 2019; 20: 4337. doi: 10.3390/ijms20184337 31487964 PMC6770454

[36] Vuilleumier N, Dayer JM, von Eckardstein A, Roux-Lombard P. Pro- or anti-inflammatory role of apolipoprotein A-1 in high-density lipoproteins? Swiss Med Wkly. 2013;143:w13781. doi: 10.4414/smw.2013.13781 23740387

[37] Singh SS, Cheung RC, Wong JH, Ng TB. Mannose Binding Lectin: A Potential for Many Human Diseases. Curr Med Chem. 2016;23(33):3847–3860. 27538693 10.2174/0929867323666160817162208

[38] Schultz H. From infection to autoimmunity: a new model for induction of ANCA against the bactericidal/permeability increasing protein (BPI). Autoimmun Rev. 2007;6(4):223–227. doi: 10.1016/j.autrev.2006.08.005 17317612

[39] Chuang HC, Chen MH, Chen YM, Yang HY, Ciou YR, Hsueh CH, et al. BPI overexpression suppresses Treg differentiation and induces exosome-mediated inflammation in systemic lupus erythematosus. Theranostics. 2021;11(20):9953–9966. doi: 10.7150/thno.63743 34815797 PMC8581436

[40] Van der Schaft DW, Toebes EA, Haseman JR, Mayo KH, Griffioen AW. Bactericidal/permeability-increasing protein (BPI) inhibits angiogenesis via induction of apoptosis in vascular endothelial cells. Blood. 2000;96:176–181. 10891448

[41] Guiot J, Njock MS, André B, Gester F, Henket M, de Seny D, et al. Serum IGFBP-2 in systemic sclerosis as a prognostic factor of lung dysfunction. Sci Rep. 2021;11(1):10882. doi: 10.1038/s41598-021-90333-0 34035374 PMC8149825

[42] DiPersio CM, Double duty for Rac1 in epidermal wound healing. Sci STKE 2007:2007(391). doi: 10.1126/stke.3912007pe33 17579242

[43] Xu SW, Liu S, Eastwood M, Sonnylal S, Denton CP, Abraham DJ, et al. Rac inhibition reverses the phenotype of fibrotic fibroblasts. PLoS One. 2009;4(10):e7438. doi: 10.1371/journal.pone.0007438 19823586 PMC2757676

[44] Gaestel M. What goes up must come down: molecular basis of MAPKAP kinase 2/3-dependent regulation of the inflammatory response and its inhibition. Biol Chem. 2013;394(10):1301–1315. doi: 10.1515/hsz-2013-0197 23832958

[45] Li LC, Li J. Geo J: Functions of galectin-3 and its role in fibrotic diseases. *J Pharmacol Exp Ther*. 2014;351(2):336–343. 25194021 10.1124/jpet.114.218370

[46] Koca SS, Akbas F, Ozgen M, Yolbas S, Ilhan N, Gundogdu B, et al. Serum galectin-3 level in systemic sclerosis. Clin Rheumatol. 2014;33(2):215–220. doi: 10.1007/s10067-013-2346-8 23912642

[47] Sundblad V, Gomez RA, Stupirski JC, Hockl PF, Pino MS, Laborde H, et al. Circulating Galectin-1 and Galectin-3 in Sera From Patients With Systemic Sclerosis: Associations With Clinical Features and Treatment. Front Pharmacol. 2021;12:650605. doi: 10.3389/fphar.2021.650605 33959016 PMC8093796

[48] Mora GF, Zubieta MR. Galectin-1 and Galectin-3 Expression in Lesional Skin of Patients With Systemic Sclerosis-Association With Disease Severity. J Clin Rheumatol. 2021;27(8):317–323. doi: 10.1097/RHU.0000000000001367 32501939

[49] Osthoff M, Jaeger VK, Heijnen IAFM, Trendelenburg M, Jordan S, Distler O, et al. Role of lectin pathway complement proteins and genetic variants in organ damage and disease severity of systemic sclerosis: a cross-sectional study. Arthritis Res Ther. 2019;21(1):76. doi: 10.1186/s13075-019-1859-1 30885245 PMC6423822

[50] Baker C, Belbin O, Kalsheker N, Morgan K. SERPINA3 (aka a-1-antichymotrypsin). Front Biosci 2007;12:2821–2835. 17485262 10.2741/2275

